# 10-Year Incidence of Diabetic Ketoacidosis at Type 1 Diabetes Diagnosis in Children Aged Less Than 16 Years From a Large Regional Center (Hangzhou, China)

**DOI:** 10.3389/fendo.2021.653519

**Published:** 2021-04-27

**Authors:** Wei Peng, Jinna Yuan, Valentina Chiavaroli, Guanping Dong, Ke Huang, Wei Wu, Rahim Ullah, Binghan Jin, Hu Lin, José G. B. Derraik, Junfen Fu

**Affiliations:** ^1^ Department of Endocrinology, Children’s Hospital, Zhejiang University School of Medicine, National Clinical Research Center for Child Health, Hangzhou, China; ^2^ Liggins Institute, University of Auckland, Auckland, New Zealand; ^3^ Neonatal Intensive Care Unit, Pescara Public Hospital, Pescara, Italy; ^4^ Department of Women’s and Children’s Health, Uppsala University, Uppsala, Sweden; ^5^ NCD Centre of Excellence, Research Institute for Health Sciences, Chiang Mai University, Chiang Mai, Thailand

**Keywords:** DKA, complications, symptoms, children, adolescents

## Abstract

**Background:**

Diabetic ketoacidosis (DKA) is a potentially life-threatening complication of type 1 diabetes (T1D), and a leading cause of death in children aged <15 years with new-onset T1D.

**Aims:**

i) to assess the incidence of DKA in children and adolescents newly diagnosed with T1D over a 10-year period at a large regional center in China; and ii) to examine the clinical symptoms and demographic factors associated with DKA and its severity at diagnosis.

**Methods:**

We carried out a retrospective audit of a regional center, encompassing all youth aged <16 years diagnosed with T1D in 2009–2018 at the Children’s Hospital, Zhejiang University School of Medicine (Hangzhou, China). DKA and its severity were classified according to ISPAD 2018 guidelines.

**Results:**

681 children were diagnosed with T1D, 50.1% having DKA at presentation (36.0% mild, 30.0% moderate, and 33.9% severe DKA). The number of patients diagnosed with T1D progressively rose from approximately 39 cases/year in 2009–2010 to 95 cases/year in 2017–2018 (≈2.5-fold increase), rising primarily among children aged 5–9 years. DKA incidence was unchanged but variable (44.8% to 56.8%). At T1D diagnosis, 89% of patients reported polyuria and 91% polydipsia. Children presenting with DKA were more likely to report vomiting, abdominal pain, and particularly fatigue. DKA was most common among the youngest children, affecting 4 in 5 children aged <2 years (81.4%), in comparison to 53.3%, 42.7%, and 49.3% of patients aged 2–4, 5–9, and ≥10 years, respectively. Children with severe DKA were more likely to report vomiting, fatigue, and abdominal pain, but less likely to report polyuria, polydipsia, and polyphagia than those with mild/moderate DKA. Rates of severe DKA were highest in children aged <2 years (51.1%).

**Conclusions:**

The number of children diagnosed with T1D at our regional center increased over the study period, but DKA rates were unchanged. With 9 of 10 children reporting polyuria and polydipsia prior to T1D diagnosis, increasing awareness of this condition in the community and among primary care physicians could lead to earlier diagnosis, and thus potentially reduce rates of DKA at presentation.

## Introduction

The incidence of type 1 diabetes (T1D) has been increasing worldwide (1–3). Diabetic ketoacidosis (DKA) is a potentially life-threatening complication of T1D that occurs with severe insulin deficiency, consisting of hyperglycemia, ketosis, and metabolic acidosis (4). This acute condition is responsible for most of the diabetes-related morbidity and mortality in affected children (5, 6). Indeed, recent data showed that DKA remains the leading cause of death in individuals aged <15 years newly diagnosed with T1D (7).

Worldwide, rates of DKA at T1D presentation vary markedly, ranging from 13% in Sweden to 80% in the United Arab Emirates (8). DKA is mainly a result of the delay in diagnosing T1D, and the concurrent failure to start appropriate insulin replacement. Therefore, DKA is a metabolic complication that is relatively easily avoidable, since it primarily reflects lack of awareness of T1D symptoms (4). Other risk factors for DKA at diabetes presentation include young age, minority ethnic groups, lower socioeconomic status, limited access to medical services, lack of medical insurance, and absence of first-degree relatives with T1D (4, 9–11). Note that, in contrast to what occurs at diagnosis, insulin omission (either inadvertently or deliberately) is the main cause of recurrent DKA (7).

DKA is associated with a large number of clinical symptoms, which include dehydration, nausea and/or vomiting, abdominal pain that may mimic an acute abdominal condition, thrombotic events, drowsiness, brain swelling, and coma (7). Although uncommon, severe brain swelling is associated with 20-30% mortality (12–14). Children with DKA may need prolonged hospital stay, with severe DKA often requiring intensive care admission. Thus, it is important to assess the epidemiology of DKA and identify the associated risk factors.

A recent study examined the incidence of T1D in 13 different regions across China (15). The authors reported a DKA rate of approximately 51.4% within 6 months of diagnosis among children aged ≤14 years, but no data from Zhejiang province were included in that study (15). Further, there are still relatively few studies comparing the clinical characteristics of patients newly diagnosed with T1D in relation to DKA severity. Therefore, we assessed the incidence of DKA among children and adolescents newly diagnosed with T1D at a regional centre in Zhejiang province over a 10-year period. In addition, we examined the clinical symptoms and demographic factors associated with the likelihood of DKA and its severity at diagnosis.

## Methods

### Ethics Approval

This study was approved by the Medical Ethics Committee of the Children’s Hospital, Zhejiang University School of Medicine. Written or verbal informed consent from individual patients was not required, as this study involved an audit of data from routine clinical practice based on de-identified data.

### Participants

Participants were all children aged <16 years diagnosed with T1D over a 10-year period (between 1 January 2009 and 31 December 2018) at the Children’s Hospital, Zhejiang University School of Medicine. The hospital is located in Hangzhou (the capital of Zhejiang province), a large city whose population increased from 6.89 million in 2010 to 7.74 million in 2018; during the same period the population of children and adolescents aged ≤17 years increase from 1.05 million to 1.33 million (+27%) (16, 17). The Children’s Hospital is one of only two National Clinical Research Centres for Child Health in China, recording 81,000 inpatient and 3.5 million outpatient visits per year. It provides specialized care for children with diabetes in Zhejiang Province.

### Study Parameters

T1D was diagnosed based on clinical and biochemical features: all patients had elevated blood glucose at presentation (a random measurement >11.1 mmol/l and/or fasting blood glucose >7.1 mmol/l), and with classical symptoms of diabetes. Further, all patients met at least one of the following criteria: 1) DKA; 2) presence of T1D-associated antibodies (glutamic acid decarboxylase, islet antigen 2, islet cell, or insulin autoantibodies); and/or 3) on-going requirement for insulin therapy.

Family and personal medical history prior to diagnosis was recorded for all children. A range of demographic information and data on clinical symptoms were collected at diagnosis from interviews with the parent(s) and patient. Clinical symptoms recorded included polyuria (excessive urination), polydipsia (excessive thirst), polyphagia (excessive eating), anepithymia (loss of appetite), weight loss, vomiting, fatigue, and abdominal pain. All patients were weighed at presentation, but height at diagnosis was not consistently measured over the study period, therefore body mass index (BMI) could not be calculated. Nonetheless, weight data were converted into standard deviation scores (SDS) as per World Health Organization standards (18, 19).

Participants underwent blood tests, and recorded parameters of interest were pH, bicarbonate, and glycated hemoglobin (HbA1c). DKA at diagnosis was defined according to ISPAD 2018 guidelines as the combination of ketosis, hyperglycemia, and acidosis (venous pH <7.3 or bicarbonate <15 mmol/L) (20). DKA was further classified as mild (venous pH <7.3 or bicarbonate <15 mmol/L), moderate (pH <7.2 or bicarbonate <10 mmol/L), or severe (pH <7.1 or bicarbonate <5 mmol/L). Antibody positivity was based on the presence of islet antigen 2 and/or islet cell autoantibodies.

Subsequently, hospitalization data (such as length of stay in hospital and the estimated cost of treatment) were obtained from hospital records.

It should be noted that all patients with DKA in our study were treated following the latest protocol as per ISPAD Clinical Practice Consensus Guidelines (20–22).

### Statistical Analyses

Data on demographic characteristics and clinical symptoms were compared between participants with and without DKA at T1D diagnosis using one-way ANOVA, Fisher’s exact tests, or non-parametric Kruskal-Wallis tests, as appropriate. Similar analyses were run comparing the three groups with DKA according to severity (i.e. mild vs moderate vs severe DKA), while differences in DKA rates among age groups (<2, 2–4.99, 5–9.99, and ≥10 years) were assessed using Fisher’s exact tests.

A generalized linear regression model was run to examine the associations between key demographic factors and the likelihood of having DKA at T1D diagnosis. The model included the following predictors: family history of T1D (yes vs no) and sex (male vs female) as categorical variables; and age at diagnosis and year of diagnosis as covariates. Results are reported as the adjusted relative risks (aRR) and respective 95% confidence intervals.

Analyses were performed using SPSS v25 (IBM Corp, Armonk, NY, USA), SAS v9.4 (SAS Institute, Cary, NC, USA), and Minitab v16 (Pennsylvania State University, State College, Pennsylvania, USA). All statistical tests were two-tailed with the significance level maintained at p<0.05. Figures were created using GraphPad Prism v8.2.1 (GraphPad Software Inc., San Diego, CA, USA).

## Results

**Figure 1:**
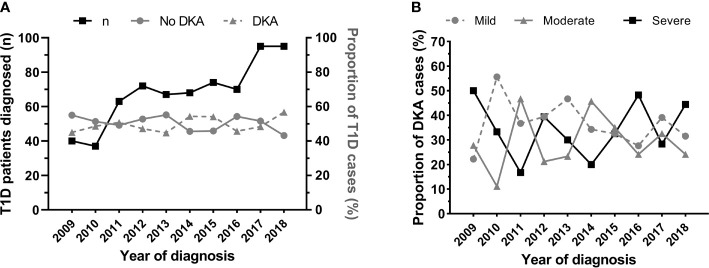
Number of patients aged <16 years newly diagnosed with type 1 diabetes (T1D) at the Children’s Hospital of Zhejiang University School of Medicine (Hangzhou, China) in 2009-2018, and rates of diabetic ketoacidosis (DKA) at diagnosis. **(A)** Number of patients newly diagnosed with T1D (black line, scale on left *y* axis) and the respective rate of DKA (solid grey line, scale on right *y* axis) and no-DKA (dashed grey line, scale on right *y* axis) at diagnosis. **(B)** Proportion of patients with mild (dashed grey line), moderate (solid grey line), and severe (black line) DKA at T1D diagnosis.

**Figure 2:**
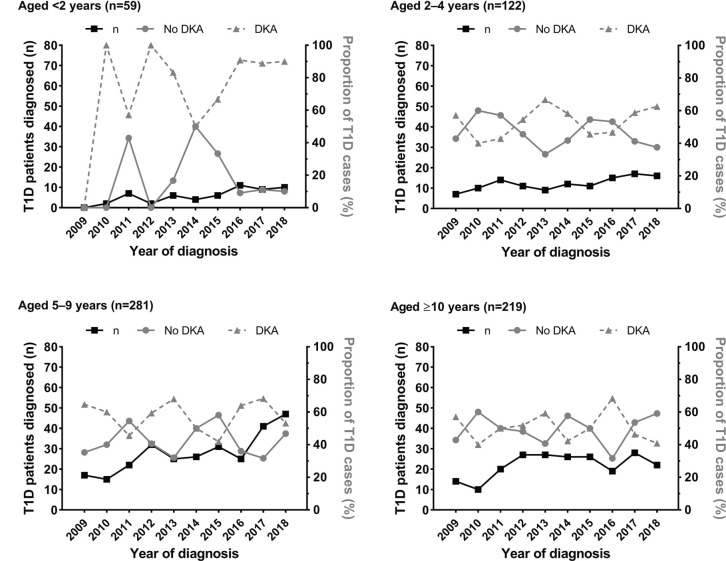
Number of patients aged <16 years newly diagnosed with type 1 diabetes (T1D) at the Children’s Hospital of Zhejiang University School of Medicine (Hangzhou, China) in 2009-2018 according to age group, and rates of diabetic ketoacidosis (DKA) at diagnosis. Data are the number of patients newly diagnosed with T1D (black line, left y axis), and the respective rates of DKA (solid grey line, right y axis) and no-DKA (dashed grey line, right y axis) at T1D diagnosis.

A total of 681 children and adolescents aged between one month and 15.8 years (314 boys and 367 girls) were diagnosed with new-onset T1D over the 10-year period, all of whom were Han Chinese. There was a progressive increase in the number of patients aged <16 years diagnosed with T1D, rising from approximately 39 cases per annum in 2009–2010 to 95 cases per annum in 2017–2018, i.e. a near 2.5-fold increase (**Figure 1A**). Notably, the increase in the number of new T1D cases occurred mostly among children aged 5–9 years (**Figure 2**).

### DKA

Half of all new cases of T1D diagnosed had DKA (n=341; 50.1%) (**Table 1**). Across the 10-year period, there was no change in the incidence of DKA (p=0.31), with variable yearly rates ranging from 44.8% to 56.8% (**Figure 1A**). Rates of DKA were also highly variable across all age groups, with no evidence of a change in incidence within these groups (**Figure 2**).

**Table 1:** Demographic and clinical data at type 1 diabetes (T1D) diagnosis in children and adolescents at the Children’s Hospital of Zhejiang University School of Medicine (Hangzhou, China), according to their diabetic ketoacidosis (DKA) status.

		All patients	No DKA	DKA	*P*-value
**n (%)**		681	340 (49.9%)	341 (50.1%)	
**Demography**	**Age (years)**	7.9 [4.7, 10.8]	8.2 [5.8, 10.9]	7.4 [3.6, 10.7]	**0.001**
	**Sex (females)**	367 (53.9%)	183 (53.8%)	184 (54%)	>0.99
	**Family history of T1D**	184 (27.1%)	104 (30.6%)	80 (23.5%)	**0.047**
**Anthropometry**	**Weight SDS**	-0.50 ± 1.15	-0.33 ± 1.09	-0.67 ± 1.18	**0.0001**
**Symptoms at diagnosis**	**Polyuria**	603 (88.5%)	307 (90.3%)	296 (86.80%)	0.19
	**Polydipsia**	620 (91.0%)	311 (91.5%)	309 (90.6%)	0.79
	**Polyphagia**	193 (28.3%)	103 (30.3%)	90 (26.4%)	0.27
	**Weight loss**	345 (50.7%)	172 (50.6%)	173 (50.7%)	>0.99
	**Vomiting**	50 (7.3%)	5 (1.5%)	45 (13.2%)	**<0.0001**
	**Anepithymia**	14 (2.1%)	3 (0.9%)	11 (3.2%)	0.055
	**Fatigue**	153 (22.5%)	19 (5.6%)	134 (39.3%)	**<0.0001**
	**Abdominal pain**	35 (5.1%)	4 (1.2%)	31 (9.1%)	**<0.0001**
**Biochemical parameters**	**pH**	7.31 ± 0.13	7.39 ± 0.04	7.22 ± 0.14	**<0.0001**
	**Bicarbonate (mmol/l)**	16.0 ± 7.0	21.1 ± 3.1	10.9 ± 6.1	**<0.0001**
	**HbA1c (%)**	12.47 ± 2.06	12.37 ± 2.14	12.57 ± 1.98	0.21
	**HbA1c (mmol/mol)**	113 ± 23	112 ± 23	114 ± 22	0.21
**Hospitalization**	**Length of stay (days)**	8 [7, 10]	8 [6, 10]	9 [7, 11]	**<0.0001**
	**Cost of treatment (RMB)^†^**	4938 [4134, 5991]	4436 [3782, 5219]	5524 [4532, 6664]	**<0.0001**
	**Cost of treatment (USD)^‡^**	765 [640, 928]	687 [586, 808]	856 [702, 1032]	**<0.0001**

Data are n (%), mean ± standard deviation, or median [quartile 1, quartile 3], as appropriate.

P-values refer to comparisons between children with and without DKA at T1D diagnosis, with p-values for statistically significant differences at p<0.05 shown in bold.

HbA1c, glycated hemoglobin; RMB, Chinese Yuan; SDS, standard deviation score; USD, United States dollars.

^†^Original recorded costs, unadjusted for inflation.

^‡^Conversion to USD accurate as of 6 January 2021.

**Figure 3:**
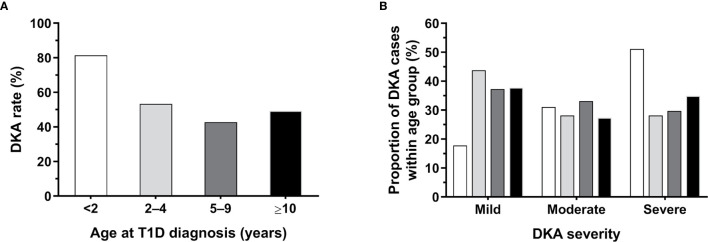
Rates and severity of diabetic ketoacidosis (DKA) among patients aged <16 years newly diagnosed with type 1 diabetes (T1D) at the Children’s Hospital of Zhejiang University School of Medicine (Hangzhou, China), according to age group. **(A)** Percentage of patients with DKA according to age at diagnosis. **(B)** Proportion of cases according to DKA severity within age group, whose corresponding bars are color coded as per panel **(A)**; the number of patients who had DKA at T1D diagnosis and data on its severity in each age group was: <2 years, n=45; 2–4 years, n=64; 5–9 years, n=118; and ≥10 years, n=106.

A slightly higher proportion of children without DKA had a family history of diabetes (27.1% vs 30.6%; **Table 1**). The likelihood of DKA was not associated with the sex of the child (p=0.77; **Table 1**), but increasing age was associated with lower risk of having DKA at diagnosis, which decreased by 4% per additional year of age [aRR 0.96 (95% CI 0.94, 0.98); p=0.0003]. Thus, on average, children with DKA were slightly younger (-0.8 years; p=0.001), and were also 0.34 SDS lighter (p=0.0001) (**Table 1**). DKA was particularly common among the youngest group of children, present in more than 4 in 5 children aged <2 years (81.4%; 48/59) at T1D diagnosis, compared to 53.3%, 42.7%, and 49.3% of patients aged 2–4, 5–9, and ≥10 years, respectively (p<0.0001; **Figure 3A**).

Overall, 89% of patients experienced polyuria and 91% polydipsia prior to T1D diagnosis. Children who presented with DKA were markedly more likely to report vomiting, abdominal pain, and in particular fatigue that was reported by ≈40% of them (**Table 1**). Conversely, reported rates of polyuria, polydipsia, polyphagia, and weight loss were similar in patients with or without DKA (**Table 1**). Children with DKA had a median hospital stay that was 1 day longer, and incurred median hospitalization costs that were 24% higher (**Table 1**).

### DKA Severity

A total of 120 children had mild DKA (36.0%), 100 children had moderate DKA (30.0%), and 113 children had severe DKA (33.9%) (**Table 2**). The proportion of children with severe DKA did not change over the study period, with a highly marked (and apparently random) variation observed from year to year (**Figure 1B**).

**Table 2:** Demographic and clinical data at type 1 diabetes (T1D) diagnosis in children and adolescents at the Children’s Hospital of Zhejiang University School of Medicine (Hangzhou, China), according to diabetic ketoacidosis (DKA) severity.

		Mild DKA	Moderate DKA	Severe DKA	*P*-value
**n**		120 (36.0%)	100 (30.0%)	113 (33.9%)	
**Demography**	**Age (years)**	7.3 [4, 10]	7.7 [4, 11]	7.4 [3, 11]	0.67
	**Sex (females)**	57 (47.5%)	61 (61.0%)	61 (54.0%)	0.14
	**Family history of T1D**	29 (24.4%)	29 (29.0%)	21 (18.6%)	0.20
**Anthropometry**	**Weight SDS**	-0.57 ± 1.21	-0.74 ± 1.07	-0.78 ± 1.24	0.36
**Symptoms at diagnosis**	**Polyuria**	113 (94.2%)	92 (92%)	86 (76.1%)	**<0.0001**
	**Polydipsia**	115 (95.8%)	97 (97%)	92 (81.4%)	**<0.0001**
	**Polyphagia**	42 (35.0%)	27 (27.0%)	19 (16.8%)	**0.007**
	**Weight loss**	75 (62.5%)	52 (52.0%)	44 (38.9%)	**0.001**
	**Vomiting**	8 (6.7%)	9 (9.0%)	27 (23.9%)	**0.0003**
	**Anepithymia**	2 (1.7%)	3 (3.0%)	5 (4.4%)	0.48
	**Fatigue**	21 (17.5%)	42 (42.0%)	68 (60.2%)	**<0.0001**
	**Abdominal pain**	8 (6.7%)	3 (3.0%)	19 (16.8%)	**0.002**
**Biochemical parameters**	**pH**	7.31 ± 0.06	7.23 ± 0.09	7.12 ± 0.17	**<0.0001**
	**Bicarbonate (mmol/l)**	14.3 ± 3.6	9.8 ± 5.2	7.7 ± 6.7	**<0.0001**
	**HbA1c (%)**	12.74 ± 2.14	12.32 ± 1.72	12.73 ± 2.01	0.23
	**HbA1c (mmol/mol)**	116 ± 23	111 ± 19	116 ± 22	0.23
**Hospitalization**	**Length of stay (days)**	8 [7, 10]	9 [8, 11]	9 [8, 11]	**0.008**
	**Cost of treatment (RMB)^†^**	4915 [4191, 5986]	5612 [4545, 6766]	6302 [5230, 8232]	**<0.0001**
	**Cost of treatment (USD)^‡^**	761 [649, 927]	869 [704, 1048]	976 [810, 1275]	**<0.0001**

Data are n (%), mean ± standard deviation, or median [quartile 1, quartile 3], as appropriate.

P-values for statistically significant differences overall at p<0.05 are shown in bold.

HbA1c, glycated hemoglobin; RMB, Chinese Yuan; SDS, standard deviation score; USD, United States dollars.

Information on DKA severity was missing for 8 of the 341 cases of new-onset T1D who had DKA at presentation.

^†^Original recorded costs, unadjusted for inflation.

^‡^Conversion to USD accurate as of 6 January 2021.

Children with severe DKA were more likely to report vomiting, fatigue, and abdominal pain than those with mild or moderate DKA (**Table 2**). Conversely, children with severe DKA were less likely to report the classical symptom of T1D at diagnosis than children with mild or moderate DKA, such as polyuria, polydipsia, polyphagia, and weight loss (**Table 2**).

The proportion of cases according to DKA severity varied among age groups, with rates of severe DKA highest in children aged <2 years (n=23/45; 51.1%) (**Figure 3B**).

Note that there were no deaths recorded in association with DKA at presentation.

## Discussion

Over the 10-year study period (2009–2018), the number of children aged <16 years newly diagnosed with T1D increased markedly at our large clinical centre in Hangzhou (China), with the observed increase recorded mostly among children <10 years of age. However, DKA rates were unchanged (although variable). Of note, half of all new cases of T1D presented with DKA, of which two-thirds were moderate or severe, and markedly more common in younger children (aged <2 years).

Stable rates of DKA (without a decline) have been reported in several countries (10, 23–25). For instance, despite an increase in the incidence of children newly diagnosed with T1D in Austria in 1989-2008, DKA rates at diabetes onset were unchanged (24). Similarly, the incidence of DKA was unchanged among children with new-onset T1D in Auckland (New Zealand) over a 15-year period (1999–2013) (10). Consistently with these findings, DKA rates at our regional centre were largely unchanged, despite the increasing number of youth diagnosed with T1D over the study period. Conversely, in a retrospective study of youth newly diagnosed with T1D in New York (USA) in 2010–2013, there was a modest decline in the rate of DKA compared to a similar study 15 years earlier (from 38% to 29%) (26). More recently, a comprehensive nationwide study in Italy also reported a slight reduction in average DKA rates at T1D diagnosis from 40.3% in 2004–2013 to 36.9% in 2014–2016, with a more marked reduction observed among children aged <5 years (27). A greater decline was reported in Saudi Arabia, from 55.1% to 32.5% between 2005 and 2015 (28). In contrast, in Colorado (USA), DKA rates increased from 41% to 58% during 2010-2017 (29), while in Malaysian children DKA rates increased from 54.5% to 66.7% in 2000–2009 (30). The reasons for these contrasting changes in DKA rates in different countries are unclear, but higher rates are generally associated with reduced community awareness of diabetes symptoms and decreased access to health care services (8, 31). Of note, our observed DKA rate (50.1% overall) was nearly identical to that reported from 13 areas across China (51.4%), although the latter figure referred to DKA up to 6 months since diagnosis (15).

In children with new-onset T1D, young age has been identified often as an important risk factor for DKA at diabetes presentation (4, 10, 27). Studies in Canada, Italy, and the UK have reported higher rates of DKA at T1D diagnosis in young children aged 0–4 years (25, 27) and <2 years (28), respectively. A meta-analysis involving 32 studies found that children aged <2 years of age had 3 times the risk of presenting with DKA compared to older children (32). In line with these studies, we observed that DKA was more common among the youngest children (<2 years of age), who also had the highest rates of severe DKA. The latter observation is in agreement with the published evidence, as DKA at diagnosis in younger children (especially <2 years of age) is usually more severe, and it is often a consequence of delayed treatment or diagnostic error (1–3, 27, 33).

In our study, children with severe DKA were less likely to report the classical symptom of T1D at diagnosis than children with mild or moderate DKA (i.e. polyuria, polydipsia, polyphagia, and weight loss). As the youngest group of children were over-represented amongst those with DKA, the reported differences might have resulted from an increased difficulty to recognize polydipsia or polyuria in very young children (10). Conversely, vomiting, anepithymia, fatigue, and abdominal pain were common symptoms among children with severe DKA. In this context, it is important to be aware of both typical and less typical symptoms of T1D in children, as early recognition of this condition allows for a timely diagnosis, which in turn minimizes the risk of DKA.

To this regard, community educational campaigns to prevent DKA have been proposed as a way of increasing awareness about T1D symptoms and related acute complications, in both parents/caregivers and health care practitioners (34–39). For instance, the ‘Parma campaign’ (Italy), which delivered posters promoting the link between enuresis, polyuria, vomiting, abdominal pain, and diabetes to schools, parents, and pediatric practices, was associated with a marked reduction in DKA incidence at diagnosis (from 78% to 12.5% over two years) (39). Recently, the Stuttgart Ketoacidosis Awareness Campaign (Germany) also focused on the typical clinical symptoms of T1D and reduced the incidence of DKA (from 28% to 16% over three years) (35). However, not all awareness campaigns have been successful in reducing DKA rates at T1D diagnosis, achieving limited or no impact (34, 36). In addition, mixed findings have also been observed. In Italy, Rabbone et al. reported 2-year nationwide data on DKA incidence among children and adolescents aged 0–18 years, soon after the initiation of a national awareness campaign (40). Surprisingly, while the overall DKA rate increased (from 38.5% in 2012–2014 to 47.6% in 2016-2017), it decreased markedly in children aged <6 years (73.8% vs 52.5%); further, in contrast to the latter observation, the rate of severe DKA actually increased among these preschoolers (from 16.6% to 21.7%) (40). Nonetheless, the key strategy for campaign success seems to be close cooperation among families, school teachers, and health care practitioners, in particular primary health care providers, such as family pediatricians as in the Italian campaign (40). In any case, our study corroborates the relevance of focusing on the classical symptoms of T1D in such campaigns (34, 40), as 89% of our patients experienced polyuria and 91% polydipsia prior to T1D diagnosis.

Apart from potential adverse neurocognitive outcomes (41, 42), DKA can also lead to death, being associated with a mortality rate <1% (8, 43). The recent study reporting on DKA at T1D diagnosis in 13 different regions across China recorded two deaths as a result of DKA among 5018 patients (15), i.e. a mortality rate of 0.04%. In our study, there were no recorded fatalities among our 681 patients.

The main limitation of the present study was the lack of key demographic data, such as access to medical services and medical insurance, and in particular socioeconomic status, as numerous studies have reported that greater socioeconomic deprivation is associated with an increased risk of DKA at T1D diagnosis (9, 11, 27, 32, 44–51). Of note, a number of studies have shown an increased risk of DKA among ethnic minorities (11, 23, 45, 50, 52), and since ethnicity and socioeconomic status are strongly intertwined (53), our homogeneous cohort consisting solely of Han Chinese patients likely mitigated some of the potential effects of socioeconomic status. Further, a key strength of our study was the relatively large number of patients examined from a large city in China over a 10-year period. In addition, all our patients presented to a single large center, so that they were all attended to according to the same treatment protocol.

In conclusion, we observed an increasing number of children being diagnosed with T1D over the study period at our large regional centre. While the rates of DKA were unchanged overall, they remained relatively high. With approximately 9 of 10 children reporting the two main clinical symptoms of T1D at diagnosis (i.e. polyuria and polydipsia), educational campaigns to increase awareness of this condition in the community and among primary care physicians could lead to earlier diagnosis, and thus potentially reduce the high rates of DKA at presentation in the region.

## Data Availability Statement

The clinical data supporting this article are not readily available because of the conditions of the ethics approval. The anonymized data on which this article was based could be made available to other investigators upon bona fide request, and following all the necessary approvals (including ethics) of the detailed study proposal and statistical analyses plan. Requests to access the datasets should be directed to Professor Junfen Fu (fjf68@zju.edu.cn).

## Ethics Statement

This study was approved by the Medical Ethics Committee of the Children’s Hospital, Zhejiang University School of Medicine. Written or verbal informed consent from individual patients was not required, as this study involved an audit of data from routine clinical practice based on de-identified data.

## Author Contributions

JF, JD, JY, HL, and BJ conceived and designed the study. JF, JY, GD, HL, KH, and WW carried out clinical assessments and collected the data. JY, HL, BJ, RU, JD, WP, GD, HL, KH, and WW contributed to data curation. JD analyzed the data. WP, VC, and JD wrote the manuscript with critical input from all other authors. All authors contributed to the article and approved the submitted version.

## Funding

This research was supported by the National Key Research and Development Program of China (No. 2016YFC1305301); Fundamental Research Funds for the Central Universities (2020XZZX002-22); the Research Fund of Zhejiang Major Medical and Health Science and Technology & National Ministry of Health (WKJ-ZJ-1804), and Zhejiang Provincial Key Science and Technology Project (LGF21H070004).

## Conflict of Interest

The authors declare that the research was conducted in the absence of any commercial or financial relationships that could be construed as a potential conflict of interest.
